# 344. Evaluation and Comparison of the Complex Outpatient Oral Antibiotics Therapy (COPAT) Program vs Oral Parenteral Antibiotic Therapy (OPAT) Program at West Virginia University (WVU) Medicine Healthcare System

**DOI:** 10.1093/ofid/ofad500.415

**Published:** 2023-11-27

**Authors:** Ololade O Okunlola, Vincenzo J Pizzuti, Jessica Johnson, Joy J Juskowich, Arif R Sarwari, Rebecca Reece

**Affiliations:** West Virginia University School of Medicine, Charleston, West Virginia; West Virginia University School of Medicine, Charleston, West Virginia; West Virginia University, Morgantown, WV; West Virginia University, Morgantown, WV; West Virginia University, Morgantown, WV; West Virginia University, Morgantown, WV

## Abstract

**Background:**

Patients with severe infections often require several weeks of antibiotic therapy. Outpatient Parenteral Antibiotic Therapy (OPAT) services decrease hospital length of stay, incidence of hospital-associated infections, healthcare costs, and increase patient recovery rates. Complex outpatient oral antibiotics therapy (COPAT) is an alternative to OPAT that transitions patients to appropriate and equally efficacious oral antibiotics. While there is extensive body of literature on OPAT, there is a paucity on COPAT. We aimed to examine the COPAT program at WVU Medicine, particularly patient demographics and clinical outcomes, and compare these to OPAT.

**Methods:**

This was a retrospective chart review of patients enrolled in COPAT and OPAT program between January 2021 to May 2022 at WVU Medicine, excluding patients who switched cohorts. From the electronic health record, both demographic and clinical variables were extracted. Descriptive analyses were done comparing demographics and clinical outcomes of OPAT, COPAT and non-intravenous drug use (IVDU) COPAT patient groups. Inferential analysis is currently underway.

**Results:**

A total of 334 charts were reviewed with 55% in OPAT program and 45% in COPAT. Demographic differences included younger age, higher tobacco and IVDU, and fewer other co-morbidities in COPAT group (Table 1). Separate analysis of non-IVDU COPAT found a similar trend of younger with fewer co-morbidities. The most common infection across groups was septic arthritis, comprising around 13% (OPAT) and 20% (COPAT) of cases. However vertebral osteomyelitis and endocarditis were higher in COPAT cohorts. Outcome findings were increased length of stay in COPAT but reduction in readmission rates compared to OPAT cohort. Increased safety interventions (22%) in OPAT compared to only 7% in COPAT, mostly due to acute kidney injury and myelosuppression. (Table 2)

**Figure:**
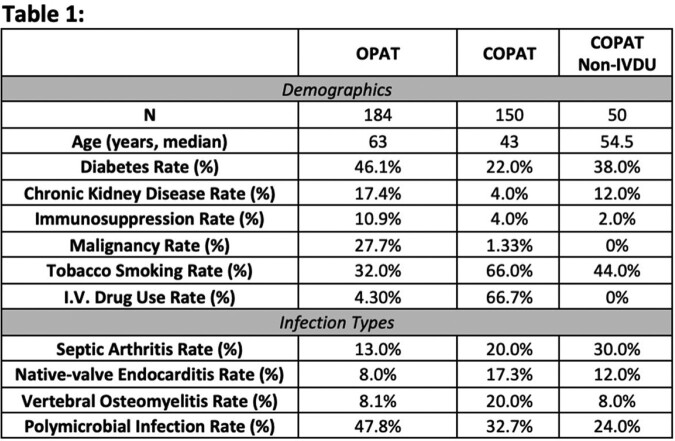

Demographics and Infection Types Seen in OPAT, COPAT and Non-IVDU COPAT Patients.

**Figure:**
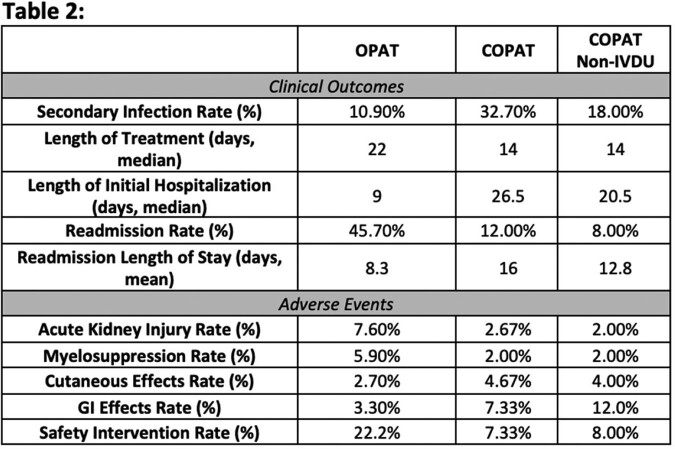

Clinical Outcomes and Adverse Events observed in OPAT, COPAT and Non-IVDU COPAT Patient Groups.

**Conclusion:**

This study demonstrated that COPAT is a suitable treatment option for both IVDU and non-IVDU patients with decreased rates of readmission and safety interventions and with lower rates of severe adverse events. While selection bias may explain differences in risk factors between OPAT and COPAT, the large variance in readmission and safety intervention rates suggests a possible causal relationship.

**Disclosures:**

**All Authors**: No reported disclosures

